# The impact of social support on the health-related quality of life of adult patients with tuberculosis in Harare, Zimbabwe: a cross-sectional survey

**DOI:** 10.1186/s13104-018-3904-6

**Published:** 2018-11-06

**Authors:** Calvin Zarova, Matthew Chiwaridzo, Catherine Tadyanemhandu, Debra Machando, Jermaine M. Dambi

**Affiliations:** 1 Department of Rehabilitation, College of Health Sciences, University of Zimbabwe, P.O Box A178, Avondale, Harare, Zimbabwe; 2 Department of Psychiatry, College of Health Sciences, University of Zimbabwe, P.O Box A178, Avondale, Harare, Zimbabwe; 3 School of Health and Rehabilitation Sciences, Faculty of Health Sciences, University of Cape Town Observatory, Cape Town, 7700 South Africa; 4 Department of Psychology, University of Cape Town, Rondebosch, Cape Town, 7701 South Africa; 5 Department of Physiotherapy, School of Therapeutic Sciences, Faculty of Health Sciences, University of the Witwatersrand, Johannesburg, South Africa

**Keywords:** Tuberculosis, Social support, Health-related quality of life, Mental health, Zimbabwe

## Abstract

**Objective:**

Tuberculosis (TB) is the second prime cause of mortality in Sub-Saharan Africa and remains a major worldwide public health problem. Unfortunately, patients with TB are at risk of poor mental health. However, patients who receive an adequate amount of social support are likely to have improved health outcomes. The study was done to establish how social support influences the health-related quality of life (HRQoL) of patients with TB in Harare, Zimbabwe. Data were collected from 332 TB patients and were analysed through structural equation modelling.

**Results:**

The mean age of the participants was 40.1 (SD 12.5) years and most were; males (53%), married (57.8%), educated (97.3%), unemployed (40.7%), stayed with family (74.4%), and reported of less than average levels of income (51.5%). Patients received the most significant amount of social support from the family. Patients also presented with lower HRQoL as they considerably reported of pain, anxiety and depression. The final model accounted for 68.8% of the variance. Despite methodological limitations, the study findings suggest that social support optimises patients’ HRQoL. Based on the patients’ responses, it was noted that patients presented with lower mental health, therefore, there is a need to develop and implement patient wellness interventions.

**Electronic supplementary material:**

The online version of this article (10.1186/s13104-018-3904-6) contains supplementary material, which is available to authorized users.

## Introduction

Tuberculosis (TB) is the second prime cause of mortality in Sub-Saharan Africa and remains a major worldwide public health problem despite the discovery of highly effective drugs and vaccines [1, 2]. The HIV/AIDS pandemic further exacerbates the burden of TB. For example, 23.1% of patients diagnosed with HIV/AIDS in Sub-Saharan Africa are reportedly co-infected with TB [1, 3]. Unfortunately, patients with TB are at risk of poor mental health and lower health-related quality of life (HRQoL) [4]. For instance, between 40 and 70% of patients with TB suffer from various common mental disorders such as depression and anxiety [4–6].

Regrettably, patients with poor mental health are unlikely to adhere to treatment regimens, and this decreases treatment efficacy [6, 7]. Further, non-compliance leads to the development of drug-resistant TB which is expensive to treat and has an increased mortality rate [8]. Therefore, poor mental health perpetuates a vicious cycle of adverse health outcomes [5, 6]. However, there is established evidence showing that patients who receive an adequate amount of social support (SS) are likely to have optimal mental health outcomes such as lower psychiatric morbidity [9] and increased HRQoL [10]. Social support is defined as the amount of both perceived and actual care received from family, friends and or the community [11]. Furthermore, SS is an essential buffer to adverse life events (e.g. diagnosis of TB), and higher SS leads to increased treatment adherence and improved treatment outcomes [12, 13]. Logically, it can be hypothesised therefore that SS may improve HRQoL of patients with adverse life events such as TB. Unfortunately, there is a lack of evidence on the mental health of TB patients residing in low resource settings such as Zimbabwe, yet the burden of the disease is quite high. The present study, therefore, sought to establish how SS influences the HRQOL of patients with TB in Harare, Zimbabwe.

## Main text

### Study design, research setting and participants

A descriptive, cross-sectional study was carried out on adult patients with TB in Harare, Zimbabwe. Participants were conveniently recruited from one low-density suburb primary care clinic and two infectious disease hospitals. These three settings were selected as they have the highest catchment of patients with TB of varying socio-economic status. Applying the following parameters; TB prevalence rate of 28.2% (p = 0.282 and q = 0.718) [2], 95% confidence interval, and expected 10% incomplete records, the minimum sample size according to STATISTICA software was 347. We recruited patients; with a confirmed diagnosis of TB according to doctor’s notes, aged ≥ 18 years, fluent in either English or Shona (a Zimbabwean native language) and had no other chronic co-morbid conditions like HIV/AIDS, among others.

### Study instruments

Social support and HRQoL were measured using the Multidimensional Scale of Perceived Social Support (MSPSS) and the EQ-5D, respectively. The MSPSS is a 12-item outcome which measures the amount of SS received from family, friends and significant other [14]. The MPSSS-Shona version is rated on a five-point Likert scale with responses ranging from strongly disagree = 1 to strongly agree = 5, and the scores are interpreted, the higher the score, the more significant the SS [15]. The EQ-5D is a generic HRQoL measuring participant’ perceived HRQoL in the following five-domains: mobility, self-care, usual activities, pain, and anxiety/depression [16]. The severity of impairments is rated on a three Likert-scale, i.e. no problem, some problem and extreme problem. The responses are log-transformed to give a utility score which ranges from zero to one, a score of one presenting perfect health status. Respondents also rate their health on a linear visual analogue scale which has a score range of 0–100 and the higher the score, the higher the HRQoL [16, 17]. The MSPSS and EQ-5D were selected for the present study as they; are standardised, generic outcomes with robust psychometrics, very brief, and have been translated and validated into Shona [14–17].

### Procedure

Institutional and ethical approval for the study was granted by the City of Harare Health Council and the Joint Research and Ethics Committee for the University of Zimbabwe, College of Health Sciences & Parirenyatwa Group of Hospitals (Ref: JREC/362/17). This study adhered to the Declaration of Helsinki ethical principles. Participants were approached as they were waiting for services at the respective research sites, and recruitment was done over 4 consecutive weeks. The principal researcher explained the study aims, and interested participants were requested to give written consent before participating. The questionnaires were self-administered to identified participants, and completed questionnaires were collected on the same day.

### Data analysis and management

Data were entered into Microsoft Excel and analysed using SPSS (version 23), STATISTICA (version 14) and STATA (Version 15). Normality was checked using the Shapiro–Wilk Test and; participants characteristics, EQ-5D and MSPSS outcomes were summarised using descriptive statistics. Correlation co-efficiencies, Chi square/Fishers’ exact tests, analysis of variance (ANOVA) and t-tests were used to determine factors influencing patients’ social support and HRQoL. Subsequently, patients characteristics (age, marital status, educational level, employment status, perceived financial status and place of residence) and MSPSS and EQ-5D were entered in the structural equation model (SEM) as endogenous and exogenous variables, respectively. The following parameters were set as a minimum criterion for model fit; Likelihood Ratio Chi squared Test (*χms*^2^)—criteria value p > 0.05, Root Mean Square Error of Approximation (RMSEA)—criteria value ≤ 0.06, Comparative Fit Index (CFI)—criteria value ≥ 0.90, Tucker-Lewis Index (TLI)—criteria value ≥ 0.90 and the Standardized Root Mean Square Residual (SRMR)—criteria value ≤ 0.06 [18, 19].

### Results

The mean age of the participants was 40.1 (SD 12.5) years. Most patients were; males (53%), married (57.8%), educated (97.3%), unemployed (40.7%), stayed in high-density suburbs (46.4%), stayed in rented accommodation (44.9%), stayed with family (74.4%), and reported of less than average levels of income (51.5%). Further, as shown in Table 1, patients received the least and highest amount of social support from friends [(mean 2.8 (SD 1.2)] and family [(Mean 3.7 (SD 1.0)], respectively, and frequencies of MSPSS responses are shown in Additional file 1. Patients considerably reported of pain, anxiety and depression (see Additional file 2 for frequencies of EQ-5D responses), and the mean HRQoL (EQ-5D-VAS) score was 51 (SD 18.1).

**Table 1:** Participants characteristics, N = 332

Variable	Attribute	Frequency, n (%)
Gender	Males	176 (53.0)
	Females	156 (47.0)
Age	Mean (SD)^a^	40.1 (12.5)
Marital status	Divorced	27 (8.1)
	Widowed	43 (13.0)
	Single	113 (34.0)
	Married	192 (57.8)
The highest level of education	Primary education	58 (17.5)
	Secondary education	217 (65.4)
	Tertiary education	57 (17.2)
Employment status	Unemployed	135 (40.7)
	Retired	15 (4.5)
	Self-employed	105 (31.6)
	Formally employed	77 (23.2)
Place of residence	High-density suburb	154 (46.4)
	Medium density suburb	94 (28.3)
	Low-density suburb	84 (25.3)
Perceived level of income	Very inadequate	71 (21.4)
	Inadequate	100 (30.1)
	Neutral	107 (32.2)
	Adequate	47 (14.2)
	Very adequate	7 (2.1)
House ownership	Owner	114 (34.3)
	Rent	149 (44.9)
	Company	21 (6.3)
	Family house	48 (14.5)
Persons staying with	Alone	26 (7.8)
	Friends	5 (1.5)
	Family	250 (75.3)
	Relatives	51 (15.4)
MSPSS scores	Friends subscale: mean (SD)^a^	2.8 (SD 1.2)
	Significant other subscale: Mean (SD)^a^	3.5 (SD 1.1)
	Family subscale: mean (SD)^a^	3.7 (SD 1.0)
	Summative scores: Mean (SD)^a^	39.7 (SD 10.6)
EQ-5D scores	Utility scores: mean (SD)^a^	0.672 (SD 0.238)
	VAS scores: mean (SD)^a^	51 (SD 18.1)

^a^Data not presented in the n (%) format

**Fig. 1:**
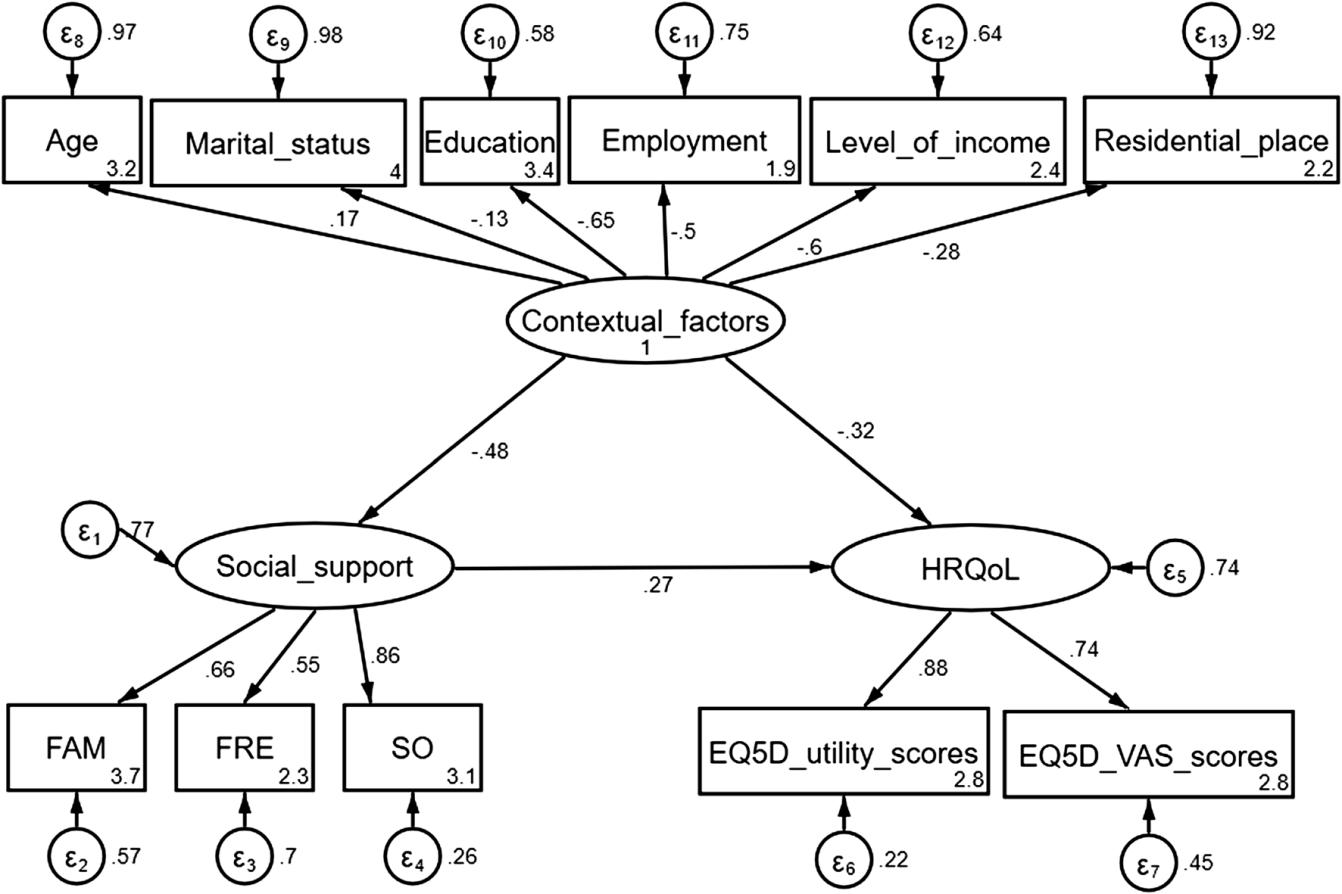
Patients’ mental health model showing the relationship between patients perceived levels of social support, HRQoL and contextual factors

The final model (Fig. 1) revealed that patients who received an adequate amount of SS had optimal/greater HRQoL, r = 0.33, p < 0.001. Further; increased age, being unmarried, lower education attainment, lower SES and residing in urban areas were associated with poorer mental health. The model displayed adequate fit, except for the Likelihood ratio, most of the goodness of fit indices were within the acceptable thresholds (see Table 2), and the model accounted for 68.8% of the variance (see Additional file 3).

**Table 2:** Model fit indices

Fit statistic	Index	Criterion for fit	Result—interpretation
Likelihood ratio	Chi squared test (*χms*^2^)	p > 0.05	*χ*^2^ (df 41) = 107.29, p < 0.001—misfit
	Normed Chi square [*χ*^2^*/df]*	*χ*^2^*/df* < 2	2.6—misfit
Population error	Root mean squared error of approximation (RMSEA)-(90% CI)	RMSEA ≤ 0.06	0.060 (0.026:0.080)—slight misfit
Information criteria	Akaike’s information criterion (AIC)	The smaller, the better	12,214.5—best fit
	Bayesian information criterion (BIC)	The smaller, the better	12,351.5—best fit
Baseline comparison	Comparative Fit Index (CFI)	CFI ≥ 0.90	0.904—good fit
	Tucker-Lewis Index (LFI)	LFI ≥ 0.90	0.871—a slight misfit
Size of residuals	Standardized root mean squared residual (SRMR)	SRMR ≤ 0.08	0.060—good fit
	The coefficient of determination (SD)	The greater, the better	0.7—good fit

### Discussion

The main finding of the present study was that patients who received an adequate amount of social support had optimal/greater HRQoL and this is congruent with previous studies [4, 9, 20]. However, patients reported lower HRQoL (mean EQ-5D VAS - 51 (SD 18.1) when compared to that of healthy urban-dwellers residing in the same research setting who previously reported a mean score of 77.5 (SD 17.4) [17]. The HRQoL outcomes were however almost like those of Zimbabwean patients with HIV/AIDS [21] which demonstrates the impact of long-term conditions on patients’ HRQoL. Invariably, pathological process/changes, e.g. persistent coughing, peripheral neuropathy, haemoptysis, fatigue and chest pain, and medication side effects such as excessive tingling sensations have been reported to contribute highly towards lower HRQoL [4, 22].

Additionally, external/environmental factors such as cultural beliefs/myths and stigma are also likely to contribute towards depression, lower self-efficacy, and lower emotional well-being which ultimately results in lower HRQoL [1, 7, 23, 24]. Evidence from a systematic review evaluating the HRQoL of South African patients with TB suggests that psycho-social burden, e.g. isolation and stigma dramatically impact patients’ HRQoL when compared to the effects of clinical symptoms [25]. This is unfortunate given that stigma precludes patients from receiving an adequate amount of SS [7, 23, 25]. Several studies concur that patients with more magnificent SS are likely; to promptly initiate diagnosis and treatment [24], comply with treatment regimens [12, 13], and have lower psychiatric morbidity [9] which will, in turn, leads to an increased HRQOL [4].

Discrepancies in the amount of SS received from family and friends is suggestive of societal stigma and or cultural influences. For instance, in the African context, it is often the responsibility of the immediate family and spouses to care for a sick relative [1]. This could explain differences in SS sources as most participants were married. Further, the present study also demonstrated the impact of contextual factors on caregivers’ mental health as reported elsewhere [5, 20, 26]. For example, patients who were; educated, formally employed and had higher levels of income had higher levels of SS and HRQoL. Patients with more financial resources are likely to afford specialist support services and likely to use medications with fewer side-effects and are thus likely to have higher HRQoL [27]. This sharply contrasts with more impoverished patients who are likely to develop anxiety and or depression because of financial pressure [24, 28]. Malnutrition and non-compliance to treatment regimens, e.g. medication intake, failure to attend scheduled follow-up appointments and lack of funds for purchasing drugs and investigative tests have been previously reported in patients residing in low-resource settings [1, 7, 24, 27, 28].

## Conclusion

The current study suggests that TB patients who receive a higher amount of social support are likely to have higher HRQoL in the Zimbabwean context. Also, given that patients reported lower mental health, there is a need to develop and implement patient wellness interventions. Further studies should utilise longitudinal and qualitative study designs and recruit patients residing in rural areas to understand the mental health of Zimbabwean patients with TB fully. Efforts should also be made to validate mental health outcomes in this population formally.

## Limitations

Although this the first large-scale study to evaluate the impact of SS on the HRQOL of tuberculosis patients in Zimbabwe the study outcomes need to be interpreted with caution given the following limitations:

- Participants and the research settings were conveniently selected. However, the setting represents the largest catchment areas of patients with TB in Harare.
- The duration of TB diagnosis and treatment were not extracted, and these may have influenced the reported mental health.
- Participants were only recruited from an urban setting thus outcomes may not be generalisable to all Zimbabwean patients given that more than 67% of Zimbabweans reside in rural areas [29].
- We only recruited participants who were proficient in either English and or Shona languages; Zimbabwe is a multilinguistic country. However, the study instruments were only adapted, translated and validated in the Shona language.
- The psychometric properties of the study instruments were not formally tested in patients with TB.
- Although we applied SEM, causality could not be inferred given the cross-sectional nature of the data.
- Confounding variables such as the length of treatment duration, type of TB, amongst others were not documented, and this may partly account for the 31.2% of the variance which was not explained by the final model.

## Additional files

**Additional file 1.** Frequencies of responses on the MSPSS, N = 332. Table denotes frequencies of responses on the MSPSS, a 12-item social support outcome measure. Responses are rated on a five-point Likert scale, ranging from strongly disagree = 1 to strongly agree = 5.

**Additional file 2.** Frequencies of responses on the EQ-5D, N = 332. Table denotes frequencies of responses on the EQ-5D, a generic health-related quality of life measure. Respondents indicate whether they had problems in with self-care, usual activities, mobility, pain/discomfort and anxiety/depression on a three-adjunct scale. Responses are rated as “no problem”, “some problem” and “extreme problem”.

**Additional file 3.** Variance explained by the model. Table denotes the variance accounted by the variables and the total model expressing the relationship between contextual factors, levels of social support and health-related quality of life.

## Abbreviations

ANOVA: analysis of variance
CFI: Comparative Fit Index
EQ-5D: EuroQol five-dimension scale
HIV/AIDS: human deficiency virus/acquired immunodeficiency syndrome
HRQoL: health-related quality of life
LTI: Tucker-Lewis Index
MSPSS: Multidimensional Scale of Perceived Social Support
RMSEA: Root Mean Square Error of Approximation
SD: standard deviation
SEM: structural equation model
SRMR: Standardized Root Mean Square Residual
SS: social support
TB: tuberculosis
