# Supplementary material for: The impact of social support on the health-related quality of life of adult patients with tuberculosis in Harare, Zimbabwe: a cross-sectional survey
Source: BMC Res Notes. 2018 Nov 6;11:795. doi: 10.1186/s13104-018-3904-6 (PMC6219075; doi:10.1186/s13104-018-3904-6)
Supplement: Supplementary file 1 — Additional file 1. Frequencies of responses on the MSPSS, N = 332. Table denotes frequencies of responses on the MSPSS, a 12-item social support outcome measure. Responses are rated on a five-point Likert scale, ranging from strongly disagree = 1 to strongly agree = 5. [file 13104_2018_3904_MOESM1_ESM.docx]

**Additional file 1: Frequencies of responses on the MSPSS,N=332**

|  | **Strongly disagree**  **n (%)** | **Disagree**  **n (%)** | **Neutral**  **n (%)** | **Agree**  **n (%)** | **Strongly Agree**  **n (%)** |
| --- | --- | --- | --- | --- | --- |
| 1. There is a special person who is around when I am in need | 56 (16.9) | 41 (12.3) | 68 (20.5) | 99 (29.8) | 68 (20.5) |
| 1. There is a special person with whom I can share joys and sorrows | 40 (12.0) | 38 (11.4) | 49 (14.8) | 102 (30.7) | 103 (31.0) |
| 1. My family really tries to help me | 30 (9.0) | 24 (7.2) | 57 (17.2) | 116 (34.9) | 105 (31.6) |
| 1. I get the emotional help & support I need from my family | 37 (11.1) | 27 (8.1) | 65 (19.6) | 95 (28.6) | 108 (32.5) |
| 1. I have a special person who is a real source of comfort to me | 46 (13.9) | 40 (12.0) | 43 (13.0) | 111 (33.4) | 92 (27.7) |
| 1. My friends really try to help me | 84 (25.3) | 67 (20.2) | 63 (19.0) | 70 (21.1) | 48 (14.5) |
| 1. I can count on my friends when things go wrong | 103 (31.0) | 76 (22.9) | 63 (19.0) | 55 (19.6) | 35 (10.5) |
| 1. I can talk about my problems with my family. | 13 (3.9) | 32 (9.6) | 52 (15.7) | 129 (38.9) | 106 (31.9) |
| 1. I have friends with whom I can share my joys and sorrows | 58 (17.5) | 63 (19.0) | 82 (24.7) | 75 (22.6) | 54 (16.3) |
| 1. There is a special person in my life who cares about my feelings. | 35 (10.5) | 42 (12.7) | 53 (16.0) | 107 (32.2) | 95 (28.6) |
| 1. My family is willing to help me make decisions | 36 (10.8) | 36 (10.8) | 59 (17.8) | 124 (37.3) | 77 (23.2) |
| 1. I can talk about my problems with my friends | 83 (25.0) | 60 (18.1) | 78 (23.5) | 76 (22.9) | 35 (10.5) |
