# Supplementary material for: The impact of social support on the health-related quality of life of adult patients with tuberculosis in Harare, Zimbabwe: a cross-sectional survey
Source: BMC Res Notes. 2018 Nov 6;11:795. doi: 10.1186/s13104-018-3904-6 (PMC6219075; doi:10.1186/s13104-018-3904-6)
Supplement: Supplementary file 2 — Additional file 2. Frequencies of responses on the EQ-5D, N = 332. Table denotes frequencies of responses on the EQ-5D, a generic health-related quality of life measure. Respondents indicate whether they had problems in with self-care, usual activities, mobility, pain/discomfort and anxiety/depression on a three-adjunct scale. Responses are rated as “no problem”, “some problem” and “extreme problem”. [file 13104_2018_3904_MOESM2_ESM.docx]

**Additional file 2: Frequencies of responses on the EQ-5D, N=332**

| **Variable** | **Attribute** | **Frequency, n (%)** |
| --- | --- | --- |
| Mobility | No problem | 214 (64.5) |
|  | Some problem | 102 (30.7) |
|  | Confined to bed | 16 (4.8) |
| Self-Care | No problems | 244 (73.5) |
|  | Some problems | 73 (22.0) |
|  | Unable to wash/dress | 15 (4.5) |
| Usual activities | No problems | 186 (56.0) |
|  | Some problems | 103 (31.0) |
|  | Unable to perform usual activities | 43 (13.0) |
| Pain/ Discomfort | No pain/ discomfort | 63 (19.0) |
|  | Moderate | 189 (56.9) |
|  | Extreme | 80 (24.1) |
| Anxiety/ Depression | Not anxious/ depressed | 156 (47.0) |
|  | Moderate | 133 (40.0) |
|  | Extreme | 43 (13.0) |
