# Supplementary material for: The impact of social support on the health-related quality of life of adult patients with tuberculosis in Harare, Zimbabwe: a cross-sectional survey
Source: BMC Res Notes. 2018 Nov 6;11:795. doi: 10.1186/s13104-018-3904-6 (PMC6219075; doi:10.1186/s13104-018-3904-6)
Supplement: Supplementary file 3 — Additional file 3. Variance explained by the model. Table denotes the variance accounted by the variables and the total model expressing the relationship between contextual factors, levels of social support and health-related quality of life. [file 13104_2018_3904_MOESM3_ESM.docx]

**Additional file 3: Variance explained by the model**

|  |  | Variance | | |  |  |  |
| --- | --- | --- | --- | --- | --- | --- | --- |
|  |  | **fitted** | **predicted** | **residual** | **R^2^** | **mc** | **mc^2^** |
| Observed variables | MSPSS Family subscale | 1.015 | .437 | .578 | .431 | .656 | .431 |
|  | MSPSS Friends subscale | 1.404 | .419 | .984 | .299 | .547 | .300 |
|  | MSPSS Significant Other subscale | 1.259 | .931 | .328 | .739 | .860 | .739 |
|  | EQ5D- Utility Scores | .0567 | .0444 | .012 | .783 | .885 | .783 |
|  | EQ5D- VAS Scores | 326.907 | 179.890 | 147.016 | .550 | .742 | .550 |
|  | Age | 156.5756 | 4.636 | 151.940 | .030 | .172 | .030 |
|  | Marital status | .731 | .0129 | .718 | .018 | .133 | .018 |
|  | Education | .346 | .146 | .201 | .420 | .648 | .420 |
|  | Employment | 1.511 | .383 | 1.128 | .253 | .503 | .253 |
|  | Level of income | 1.085 | .392 | .693 | .361 | .601 | .361 |
|  | Place of residence | .672 | .0532 | .619 | .079 | .281 | .0791 |
| Latent  variables | Social support | .437 | .102 | .335 | .239 | .484 | .234 |
|  | HRQoL | .0444 | .011 | .0329 | .258 | .508 | .256 |
|  |  |  |  |  |  |  |  |
| Overall variance explained by the model | | | | | **.688** |  |  |

***Key:*** *HRQoL- health-related quality of life; VAS- visual analogue scale: MSPSS- Multidimensional Scale of Perceived Social Support*
